# Improvement of Cottonseed Oil and Fatty Acids Through Introgression Breeding in Upland Cotton

**DOI:** 10.3390/plants14193078

**Published:** 2025-10-05

**Authors:** Savyata Kandel, Francisco Omar Holguin, Claudia Galvan, Yi Zhu, Jane Dever, Carol Kelly, Derek Whitelock, Jinfa Zhang

**Affiliations:** 1 Department of Plant and Environmental Sciences, New Mexico State University, 945 College Drive, Las Cruces, NM 88003, USA; savuknde@nmsu.edu (S.K.); frholgui@nmsu.edu (F.O.H.); cjgalvan@nmsu.edu (C.G.);; 2 Plant and Environmental Sciences Department, Clemson University, Florence, SC 29505, USA; jkdever@clemson.edu; 3 Texas A & M Agrilife Research and Extension Center, Lubbock, TX 79403, USA; cmkelly@ag.tamu.edu; 4 Southwestern Cotton Ginning Research Laboratory, Las Cruces, NM 88005, USA; derek.whitelock@usdas.gov; 5 School of Agriculture, Tennessee Tech University, Cookeville, TN 38505, USA

**Keywords:** cottonseed oil, fatty acids, introgression breeding, upland cotton

## Abstract

Upland cotton is an important fiber and oilseed crop. Cottonseed produces approximately 15% of farm gate value in cotton production. Therefore, improvement of cottonseed oil can significantly increase the economic return of cotton production with the same land use and investment. However, genetic variation in cottonseed oil is highly limited within upland cotton, limiting the genetic gain in cottonseed oil. Introgression breeding can alleviate this bottleneck effect by introducing desirable genes from Pima to Upland cotton. The objective of this study was to evaluate introgression lines (ILs) for better cottonseed oil. A population of 590 ILs, developed from a cross between Acala 1517-99 and Pima, was grown in Las Cruces, NM in 2022 which was used for the fatty acid methyl ester analysis through gas chromatography. There was a high level of variation in cottonseed oil and fatty acids. In the biplot, cottonseed oil was positively correlated with oleic acid and negatively related with palmitic acid. The cluster analysis identified a group of ILs with the highest average oil and oleic acid. As a result, ILs with better oil profiles were identified for further testing and analysis toward the development of high-quality cotton varieties with higher and better oil.

## 1. Introduction

Most cultivated tetraploid cotton, *G. hirsutum* and *G. barbadanse,* are primarily grown for fibers to produce textiles and clothing. However, cotton is also an important oilseed crop. The remaining byproduct after the extraction of oil is cottonseed meal, rich in protein, and can be used as animal feed and organic fertilizer. Cotton, the fifth oil-producing crop, is one of the major sources of vegetable oil as cottonseed contains 17–35% oil. Cottonseed oil is also known as hydrogenated oil because of the balanced amount of stearic, palmitic, and oleic acid which ensures stable frying without additional processing [1]. It is good for frying as it imparts a toasted aroma to the fried products. Because of its neutral flavor, it is mostly used for making edible products as it maintains the flavor of the items to be cooked. Besides the food sector, it is also used in cosmetic products, specialty soaps, detergents, and the biodiesel industry.

One tablespoon of cottonseed oil provides 120 calories, 3.5 g saturated fatty acids along with vitamin A, K, antioxidants, and amino acids [1]. The high amount of natural antioxidants and alpha tocopherols (35 mg per 100 g) prolong the shelf life of cottonseed oil [2]. Its nutritional components and quality attract much attention to increasing worldwide demand for vegetable oil. The global cottonseed oil market size was valued at around USD 5.52 billion in 2024 and projected to reach USD 8.03 billion by 2034 at a rate of 3.82% compound annual growth rate [3]. So, there is a significantly rising market for cottonseed oil due to the growing awareness about the health benefits of cottonseed oil and the rising standard of living.

The fatty acid composition largely determines the quality and nutritional value of plant oils. Cottonseed oil has around 65–70% unsaturated fatty acids and 26–35% saturated fatty acids with linoleic acid (55%) being the most prominent followed by palmitic acid (26%), then oleic acid (15%), and finally stearic acid (2%). The higher percentage of palmitic acid contributes towards the oxidative stability during frying which compensates the instability of unsaturated fatty acids [4]; however, it may be responsible for increasing the risk of cardiovascular diseases [5]. Therefore, consumers want a reduced level of these acids in processed foods. Among saturated fatty acids, stearic acid is classified as a desired dietary component compared to palmitic and myristic acids, as it may lower LDL cholesterol. In addition, stearic acid enhances the solidity and plasticity required for margarine and shortening as well as increases oxidative stability during frying. Therefore, stearic acid is considered a desired saturated fatty acid to improve the oil quality. Because of oxidative instability, unsaturated fatty acids upon longer deep-frying convert into short-chain hydroperoxide, aldehydes, and keto derivatives which are responsible for an off-type flavor and short shelf life [6]. Oleic acid has a similar low-density lipoprotein (LDL) lowering effect as linoleic acid; however, it is comparatively stable towards oxidative decomposition at higher temperatures. Oils with high oleic acid impart improved cooking stability for deep frying and are beneficial to human health [5,6]. Hence, it is requisite to develop cotton varieties with reduced palmitic acid and linoleic acids while correspondingly increased oleic and stearic acids.

The cottonseed oil content is affected by the genotype, environment, and their interactions. It varies among different growing seasons, locations, and years [7,8]. The interactive effect of genotype and environment depends on environmental factors (water, soil, temperature, and light) and the relative contribution of parent genotypes on the trait [9]. However, the fatty acid composition is mostly determined by the genotype of the developing embryo of cottonseed [10,11]. Generally, the quality of oil and fatty acids depends on the quality of seeds and plant species or varieties. There are examples of the effect of environment and genotype on cottonseed oil. For instance, high-quality oil is obtained from seeds growing in the dry season while lower quality oil is obtained from seeds growing in wet weather in the field or stored with high moisture. Furthermore, diploid cotton, *G. arboreum* and *G. herbaceum*, have a lower oil percentage in comparison with new world tetraploid cotton, *G. hirsutum* and *G. barbadense*, because the diploid cotton produces smaller seeds [12,13].

A sufficient genetic variability occurred in cottonseed oil within the *Gossypium* genus which could be used for making genetic gains [14]. However, low genetic diversity among the elite upland cotton (*G. hirsutum*) as a result of extensive selection within the species toward improving lint yield and quality limits the chance for breeding within the species to improve seed oil content (SOC) [15]. Therefore, *G. hirsutum* can be crossed with *G. barbadense* followed by selection to improve oil accumulation by overcoming the bottleneck of low genetic variation within existing upland cotton. *G. barbadense* is often used as an important germplasm donor to improve yield [16], fiber quality [17], disease resistance, and oil content in *G. hirsutum* [18]. Many QTL alleles from *G. barbadense* were illustrated as very favorable to improve SOC in introgressed *G. hirsutum* lines [19]. Therefore, introgression breeding between *G. hirsutum* and *G. barbadense* may hugely enhance the cottonseed oil quantity and quality. The interspecific introgression line population of *G. hirsutum* and *G. barbadense* has been frequently utilized to determine the genomic regions associated with interested quantitative traits in cotton [20]. There were some studies of using the *G. barbadense* in the background of *G. hirsutum* to improve SOC. Wu et al. [21] and Yu et al. [22] verified the importance of *G. barbadense* to improve SOC in the background of *G. hirsutum* while studying the correlations between cottonseed traits. Also, Shockey et al. [23] confirmed that *G. barbadense* accessions have high SOC characteristics among cotton germplasm resources. Thus, the genetic variation in cottonseed oil in the *Gossypium* genus can be used to genetically improve the oil content and quality leading to the breeding of high and improved oil cotton varieties while keeping its desired yield and fiber quality at acceptable levels [24,25].

Most of the cotton research has been conducted on fiber yield and quality; however, very little research has been performed on cottonseed oil while discussing the variability and correlation in the fatty acid profile in cottonseed oil. This gap in the research requires the screening of the population of cotton genotypes for higher oil content and improved fatty acid profile. So, it would be helpful to develop cotton varieties with improved oil and fatty acid composition which increase the economic return of cotton production for cotton producers without additional land use and investment. This would significantly impact cotton growers and subsequently the US economy. Therefore, this study was conducted for the screening of the population of cotton introgression lines for higher cottonseed oil and improved fatty acid profile. The objectives of this study were to (1) evaluate a large introgression line (IL) population for cottonseed oil and major fatty acids; and (2) select promising ILs with high oil and improved fatty acid profiles to contribute to the development of high-quality cotton varieties with higher and better oil content.

## 2. Results

### 2.1. Cottonseed Oil and Fatty Acids

**Figure 1:**
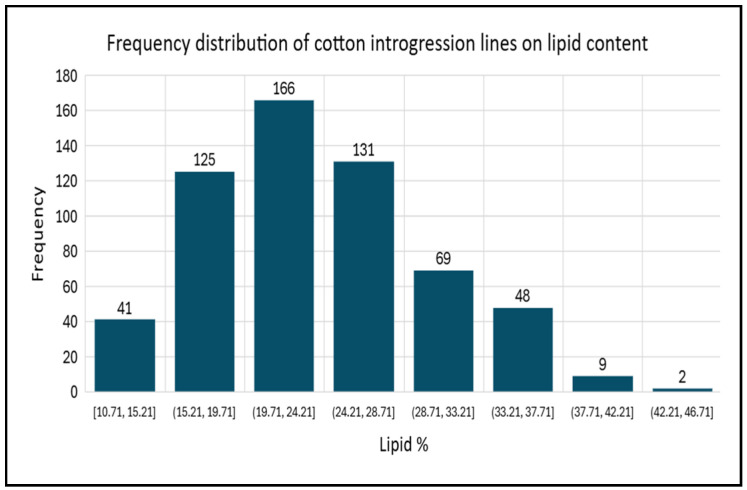
Frequency distribution of cotton introgression lines on lipid content.

The relative value of mean and the range of oil and individual fatty acids of cotton introgression lines were presented in Table 1 and Figure 1. The average value for cotton seed oil content (SOC) was 24.01% and ranges from 10.71% to 43.10%. It indicated that a greater genetic variation existed among the studied cotton introgression lines. The SOC has a high level of unsaturation (68.92%) ranging from 57.24 to 73.20% whereas the average value of saturated fatty acid was 30.92% ranging from 27.20 to 42.76%. Myristic (C14:0), palmitic (C16:0), stearic (C18:0), arachidic (C20:0), behenic (C22:0), and lignoceric (C24:0) acids were found as saturated fatty acids. In addition, monounsaturated fatty acids were palmitoleic (C16:1) and oleic (C18:1) acids while polyunsaturated fatty acids were linoleic (C18:2) and linolenic (C18:3) acids. About 10 fatty acids were identified as percentages of the total fatty acid in cottonseed oil, but not all introgression lines had all fatty acids.

**Figure 2:**
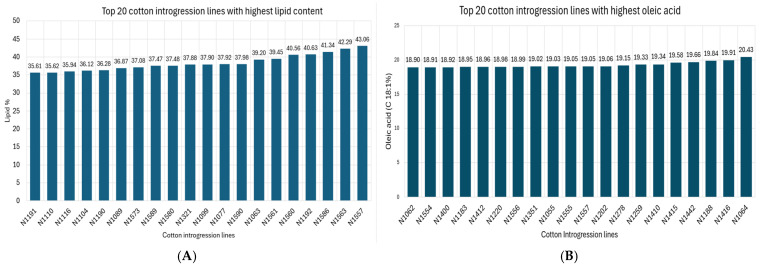

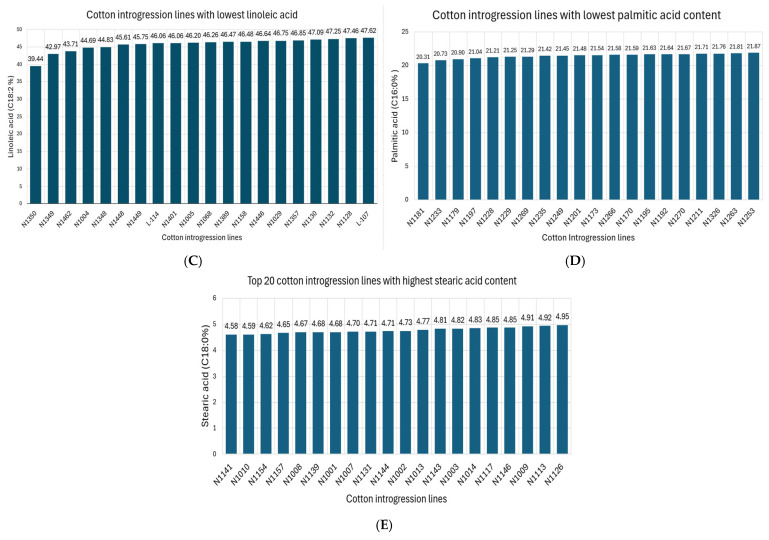
Top 20 cotton introgression lines; (**A**): highest lipid content; (**B**): highest oleic acid; (**C**): lowest linoleic acid; (**D**): lowest palmitic acid; (**E**): highest stearic acid.

The principal fatty acid is linoleic acid (39.44–56.10%) followed by palmitic acid (20.3–30.0%), oleic acid (13.4–20.4%), and stearic acid (0.8–5.8%) in our study (Table 1). Edible oil with a high proportion of oleic acid represents healthy oil due to the low content of saturated fatty acids and no need for chemical hydrogenation. The average value of oleic acid is 17.12% in our study (Table 1). The increase in oleic and stearic acids imparts the required functional properties on modified oil. Even though the higher level of palmitic acid is important to the stability of oil and the solidity of its hydrogenated derivatives, it is nutritionally undesirable. So, it should now be possible to reduce palmitic acid in cottonseed oil without compromising performance [6]. Top 20 ILs with the highest lipid, oleic acid and stearic acid and the lowest linoleic acid and palmitic acid are shown in Figure 2.

### 2.2. Principal Component Analysis

**Figure 3:**
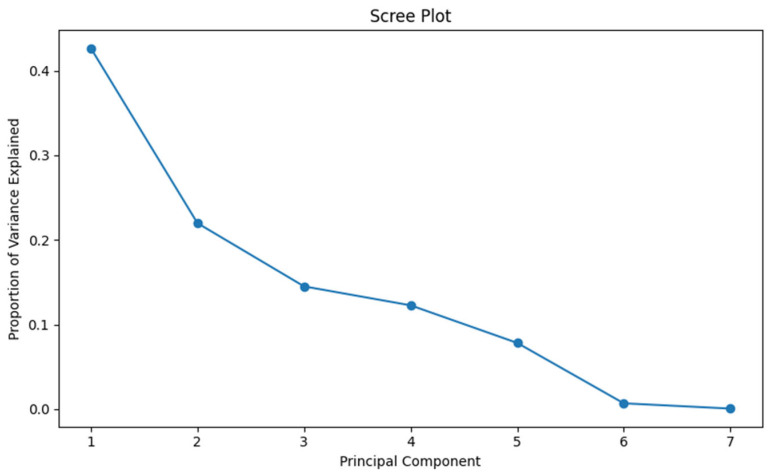
Scree plot of principal component analysis using R-matrix.

The total variance was divided into seven principal components (PCs) as shown in Table 2. The first 3 PCs out of the total 7 PCs displayed more than one eigenvalue. Furthermore, the scree plot (Figure 3) demonstrated the proportion of variance with all principal components where natural break occurs even after the first two principal components. PC I displayed the highest variability of 42.7% with the eigenvalue of 1.728 while minimum variability was observed in PC VI and PC VII with the eigenvalues of 0.224 and 0.048, respectively. PC I and PC II exhibited about 64.7% cumulative variability (Table 2). The other PCs cover only a little information about datasets such as PC III, PC IV, and PC V, having 14.5%, 12.3%, and 7.8% share of total variability, respectively (Table 2).

After performing principal component analysis (PCA), traits/genotypes were exposed to biplot analysis to see the association among them. Even though seven different variables had various contributions in total variability, the first two PCs share maximum variation to the dataset. Therefore, the two-dimensional representation was illustrated by using the first two PCs for further explanation.

### 2.3. Biplot Analysis

**Figure 4:**
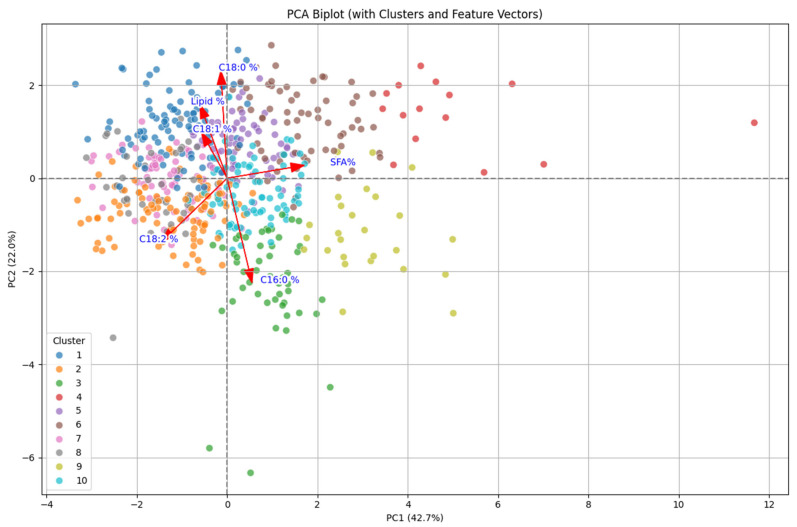
Plot of first two principal components for contribution of traits and cotton introgression lines.

Biplot analysis is used to show the association among traits/genotypes and principal components where variables were represented on the plot in the form of vectors. The share of each variable to the total variation is expressed through the relative distance of the variables from the origin to the principal components. Lipid and oleic acid (C18:1) showed minimum differences as they were close to the origin whereas linoleic (C18:2), stearic (C18:0), palmitic acids (C16:0), and saturated fatty acids displayed maximum differences as they were at a greater distance from the origin (Figure 4).

In biplot analysis, PC I is positively related to lipid, oleic, and linoleic acids (Table 3). Also, it is positively correlated with stearic acids which is a desirable saturated fatty acid. However, it is negatively related to palmitic acid. On the other hand, PC II is highly and positively correlated with lipid content, stearic acid, and oleic acid as compared to PC I but negatively with palmitic and linoleic acids (Table 3). Among seven principal components, PC I and PC II showed the maximum contribution to the total variation, and these two PCs are mainly related to lipid, oleic, stearic, and linoleic acids, so these four traits are responsible for contributing maximum variation in the dataset. In addition, genotypes in PC I and PC II have maximum variability, implying that the selection of those genotypes should be performed to improve oil content and quality.

### 2.4. Cluster Analysis

**Figure 5:**
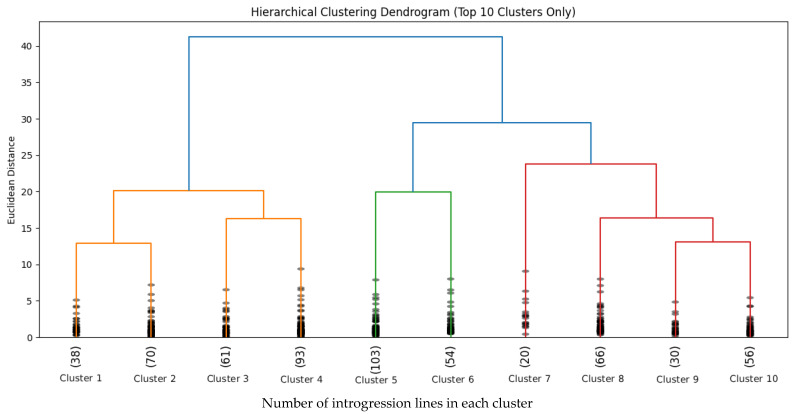
Dendrogram resulting from hierarchical cluster analysis of cotton introgression lines.

Cluster analysis is one of the important and effective methods to identify and establish a structured relationship between different genotypes. Also, it establishes a hierarchical classification among those genotypes. The ward method was used for cluster analysis of 590 cotton introgression lines in our study. Cluster analysis (dendrogram) for oil, four major fatty acids (palmitic, oleic, stearic, and linoleic acids), saturated fatty acids, and a ratio of saturated-to-unsaturated fatty acids is shown in Figure 5. Based on these characteristics, the 590 cotton introgression lines were grouped into different clusters depending on the cut-off point. At 10 Euclidean distances, those 590 introgression lines were grouped into ten different clusters (Figure 5).

Among ten different clusters, cluster 5, 4, 2, 8, and 3 are major clusters comprising 103, 93, 70, 66, and 61 cotton introgression lines, respectively, while cluster 10, 6, 1, 9, and 7 are comparatively smaller with 56, 54, 38, 30, and 20 introgression lines, respectively (Figure 5). Genotypes included in one cluster were significantly different from genotypes grouped in another cluster regarding cottonseed oil and major fatty acid content. Cluster 1 has the highest average lipid percentage and higher content of stearic and oleic acids and a comparatively lower amount of linoleic acid and saturated fatty acids as well as a lower ratio of saturated to unsaturated fatty acids (Table 4). As compared to cluster 1, cluster 2 contains introgression lines with lower average value of oil, oleic acid, stearic acid, and a lower ratio of SFA to USFA and higher linoleic acid and palmitic acid (Table 4). Like cluster 2, cluster 8 has similar values of stearic acid, saturated fatty acid, and the ratio of SFA-to-USFA. However, it has the highest oleic acid and comparatively lower lipid content. Furthermore, cluster 6 also has the higher average lipid percentage, stearic and oleic acid, and lower linoleic acid. However, it has higher saturated fatty acid content and a higher ratio of saturated-to-unsaturated fatty acids as compared to clusters 1 and 2. Among ten clusters, cluster 9 has the lowest mean value of lipid and lower stearic, oleic and linoleic acids, as well as higher saturated fatty acids and a higher ratio of SFA to USFA. To increase cottonseed oil content along with better oil quality, selection of cotton introgression lines should be performed from clusters 1, 2, and 8. If the objective is solely increasing the oleic acid, then we could make the selection of introgression lines from cluster 8.

Among 590 cotton introgression lines, N1063 (39% oil, 19% oleic, 4% stearic, 25% palmitic, 53% linoleic, and 0.4 of SFA/USFA) and N1190 (36% oil, 19% oleic, 4% stearic, 23% palmitic, 50% linoleic, and 0.42 of SFA/USFA) performed better in terms of higher and better oil quality. These two introgression lines were grouped in cluster 1 in the dendrogram (Table S1) and have higher oil percentages as well as higher oleic and stearic acids which are desirable fatty acids to make the oil quality better in terms of cooking and health context. However, those genotypes have a lower content of linoleic acids and the ratio of saturated-to-unsaturated fatty acids which is desirable to make the oil more stable.

## 3. Discussion

As dietary fatty acid composition has effects on human health, lipid composition is important for the quality of oils. Some SFAs showed detrimental effects, while intake of MUFAs and PUFAs minimized the risks of cardiovascular and other metabolic diseases, consequently improving the health span [26]. Saturated fatty acids have no double bonds between the carbon atoms, which means they are fully saturated with hydrogen atoms. Also, they are chemically least reactive and have a higher melting point than unsaturated fatty acids of the same chain length. They are typically solid at room temperature and are found in higher amounts in animal products such as meat and dairy and some plant oils. Mainly, palmitic and stearic acids comprise the saturated fatty acids in cottonseed oil. Hamza et al. [27] found average values of 23.0 to 25.5% among cotton genotypes in studying saturated fatty acid composition in Egyptian cotton. According to the O’Brien et al. [28] results, cottonseed oil has sufficient saturated (palmitic ~22%, stearic ~3%) and unsaturated (oleic ~22%, linoleic ~52%) fatty acids to make it a relatively stable vegetable oil as well as heart healthy oil, respectively. In addition, Dowd et al. [29] observed that the concentration of palmitic acid is higher in cottonseed oil (~24%) than in many other vegetable oils and Hall [30] reported an average value of 25.73% palmitic acid in upland cotton. These values are in accordance with our studies. Wan et al. [31] and Shekar et al. [32] observed a mean value of 2.1% of stearic acid in cottonseed while Sharma et al. [33] found 2.38% of average stearic acids, which varied from 0.81 to 3.97%. However, we found 3.38% of the average stearic acid, which varies from 0.8 to 5.8% in our study. This variation in stearic acid among cotton introgression lines may be due to the genetic characteristics of cotton introgression lines in fatty acid composition.

Unsaturated fatty acids have one or more double bonds in the fatty acid chain. Depending on the number of double bonds, they can be classified as monounsaturated (one double bond) or polyunsaturated (more than one double bond). Unsaturated fats are usually liquid at room temperature but begin to solidify at a low temperature as compared to saturated fatty acids. They are found in higher amounts in plant-based oils like olive oil, canola oil, various nut oils, and fatty fish. They are considered healthier fats because they can improve blood cholesterol levels, decrease inflammation, and provide essential fatty acids that our body needs but cannot produce on its own. Unsaturated fatty acids are more reactive in comparison with saturated fatty acids as their reactivity increases with the increase in the number of double bonds. Diets high in saturated fats have been linked to increased risk of heart disease and high cholesterol levels. Unsaturated fats, especially polyunsaturated fats, have been associated with reduced risk of heart disease and other health benefits when consumed in moderation. In summary, it is important to have a balanced intake of fats in our diet, emphasizing unsaturated fats over saturated fats to promote overall health and well-being. A particular ratio of saturated and unsaturated fatty acids in cottonseed oil gives it a peculiar taste and cooking quality. Oleic and linoleic acids collectively constitute the unsaturated fatty acids in cottonseed oil. Lukonge et al. [34] found 70.2 to 74.9% of unsaturated fatty acids in the evaluation of 24 upland cotton genotypes for fatty acid profile which our study is following. Likewise, Lawhon et al. [35] observed 70.0 to 79.6% of unsaturated fatty acids while studying the seed composition of eight of each glanded and glandless cotton genotypes. Oleic acid, the greasy materials in cottonseed oil, varied over 13.39 to 20.40% as (Table 1) in our study, which are comparable with most varieties of the cottonseed oils investigated in Sharma et al. [33]. We found mean oleic acid contents of 20.21% (Table 1) which are in accordance with Dowd et al.’s findings [29]. The average linoleic acid in our study among introgression lines was 51.04% and varied from 39.00 to 56.10%. These results follow the findings of Mathaus and Ozcan [36] where they observed 53.2% of linoleic and γ-linolenic acids in cottonseed.

Statistically, lipid and various fatty acids analyses showed differences among cotton introgression lines for all traits. As our study included the tenth-generation population obtained from the interspecific cross between *Gossypium* species, several lines were showing variation in oil and fatty acid content because of transgressive segregation. So, there is a great genetic variation in cottonseed oil and major fatty acids among the introgression lines, illustrating the possibilities of obtaining genetic improvement by applying the selection. It shows that breeding within this population would improve the oil content and composition in cottonseed, thereby enhancing the seed quality of cotton.

Principal component analysis is conducted to identify the relationship among genotypes and the studied traits. The total variance was divided into seven principal components. Data in each component with an eigenvalue more than one determined at least 10% of the variation according to Ullah et al. [37]. Therefore, higher eigenvalues were taken as major representative attributes in principal components. In our study, the first 3 PCs out of the total 7 PCs displayed more than one eigenvalue. As the first two principal components explained the 64.7% cumulative variability, those two principal components were retained for biplot analysis. PC I is positively related to lipid, oleic, linoleic, and stearic acids. This refers to the association of the first principal component to unsaturated fatty acids and lipid content in cottonseed oil. So, genotypes having high PC I value have higher linoleic and oleic acids as well as high oil content. By selecting the genotypes having a higher value of PC I, we could increase lipid content and oleic acid as well as linoleic acid. On the other hand, PC II is highly and positively correlated with lipid, stearic, and oleic acids while negatively with palmitic and linoleic acids. This refers to the fact that PC II is positively related to higher lipid and good fatty acid content while negatively with bad fatty acids indicating that cotton genotypes with high PC II value are beneficial for improving the quality and quantity of cottonseed oil. Therefore, selecting the genotypes having a higher value of PC II indicates that those genotypes have a high content of lipid, stearic, and oleic acids while less value of linoleic and palmitic acids. So, these fatty acid compositions make the cottonseed oil healthier and more stable while cooking. Thus, we can select the cotton introgression lines from PC II to increase cottonseed oil content and quality to contribute to the development of high oil containing quality cotton varieties. As PC I and PC II are highly related to lipid content, oleic, and linoleic acids, genotypes in PC I and PC II should opt for selection as they have maximum variability (Figure 4). So, those genotypes could be utilized in future breeding programs to develop improved cotton varieties. The principal component analysis revealed the variation in lipid and various fatty acids in cottonseed oil among the cotton introgression lines which can be utilized in breeding programs to obtain the desirable genotypes.

In the cluster analysis, 590 cotton introgression lines were grouped into ten different clusters at 10 Euclidean distances in the dendrogram obtained from the hierarchical cluster analysis (Figure 5). The cotton introgression lines grouped into one cluster were significantly different from the introgression lines included in another cluster. Among 10 clusters, Cluster 1 has the highest average lipid content and higher stearic and oleic acid but comparatively lower linoleic acid and saturated fatty acid content as well as a lower ratio of saturated-to-unsaturated fatty acids. However, cluster 2 contains introgression lines with lower oil, oleic, and stearic acid content and a lower ratio of SFA-to-USFA while higher linoleic and palmitic acid content as compared to cluster 1. From hierarchical cluster analysis, it was found that selecting the cotton introgression lines from cluster 1 is best to increase lipid content, oleic acid, and stearic acid while lowering the amount of linoleic acid at the same time. So, we can choose the lines from cluster 1 to breed cotton varieties with higher and better oil quality. Besides cluster 1, we can select cotton introgression lines from cluster 2 to improve cottonseed oil quality as well as quantity. Among 590 cotton introgression lines, N1063 (39% oil, 19% oleic, 4% stearic, 25% palmitic, 53% linoleic, and 0.4 of SFA/USFA) and N1190 (36% oil, 19% oleic, 4% stearic, 23% palmitic, 50% linoleic, and 0.42 of SFA/USFA) performed better in terms of higher and better oil quality which were grouped into cluster 1 in the dendrogram (Table S1).

## 4. Materials and Methods

### 4.1. Field Trials and Seed Samples

The cotton introgression lines used in this research are 10th generation progenies (F10) developed from a cross between Acala 1517-99 and a Pima cotton parent at New Mexico State University. A total of 1500 introgression lines (ILs) were grown in Leyendecker Plant Science Center, NMSU in 2022. The experimental design was an augmented design with 4 checks (3 Acala and 1 Pima including parents) that were replicated in each block as randomized complete block design. In total, 20 bolls per sample were harvested and ginning was performed to obtain fiber and fuzzy seeds. Acid-delinted cottonseeds were used for fatty acid methyl ester analysis.

### 4.2. Fatty Acid Methyl Ester Sample Preparation

A total of 590 cotton samples were subjected to FAME (Fatty acid methyl ester) analysis in 2023 and 2024. Fifty grams of seed from each sample were grinded by a coffee grinder to make fine powder. The dry weight of cotton samples was taken by subtracting the weight of vial with sample kept in oven at 35-degree Celsius overnight to the weight of vial with sample before kept in oven.

A 50 μL of internal standard solution (C13:0 ME, at 10 mg/mL) and 200 μL of chloroform/methanol (2:1 *v*/*v*) was added to 5–10 mg of cotton tissue in a 2 mL GC vial. A trans-esterification reaction was catalyzed by the addition of 300 μL of 0.6 M hydrochloric acid/methanol followed by vertexing and the mixture were kept on a preheated block at 85 degrees Celsius for 1 h and was left to cool down for at least 15 min. The resulting trans-esterified fatty acids were back extracted into 1 mL of hexane and vortexed well, then kept undisturbed for at least 1 h. Total FAMEs were separated into 800 μL of hexane and analyzed through a Varian 3900 gas chromatograph (GC) by ColeParmer (Vernon Hills, IL, USA). The GC system was fitted with a split/splitless injector, automated sampler, a flame-ionization detector (FID), and DB-Wax capillary column (0.25 mm internal diameter × 30 m × 0.25 μm film thickness). FAME samples of 1 μL were injected at a 10:1 split ratio with an inlet temperature of 250 °C. Helium was used as carrier gas and controlled in constant flow mode at a linear velocity of 1 mL/min. The oven was programmed to start at 100 °C for 1 min, then the temperature was ramped at 25 °C/min up to 200 °C and held for 1 min; again, the temperature was ramped at 5 °C/min up to 250 °C, which was held for an additional 7 min. The flame ionization detector was programmed with the following settings: 280 °C, 450 mL/min zero air, 40 mL/min H2, and 30 mL/min helium. External calibration was used to generate a standard curve from a serial diluted FAME standard calibration mix C8:0–C24:0 (NuChek-Prep GLC 461C). The resulting standard curves for each FAME standard, and the internal standard (Methyl tridecanoic acid; C13:0 ME) were used to quantify peaks within samples.

### 4.3. Statistics

All the data were subjected to Microsoft Word Excel to analyze the descriptive statistics. The frequency distribution of genotypes was performed in Excel and presented in graphical form. In these plots, the width of the component band reflects a variation due to genetics. The principal component and cluster analyses were conducted in R-studio 4.5.1 to identify relationships among observed traits and genotypes in cotton.

## 5. Conclusions

There is an existence of great genetic variation among cotton introgression lines for cottonseed oil and different fatty acid content. The variation among accessions was found for stearic, palmitic, linoleic, and oleic acids in cottonseed oil. This variation is associated with genetics. So, selection may be possible to improve oil quality and quantity of cottonseed in the population of cotton introgression lines. Stearic acid is negatively correlated with palmitic acid, while lipid content is positively correlated to oleic acids; and therefore, breeding for increased lipid and oleic and stearic acids while reduced palmitic acid is possible through introgression breeding. Moreover, lipid is negatively correlated with linoleic acids in PC II. So, breeding for increased oleic acid, stearic acid, and lipid content as well as consequently reduced linoleic acids at the same time might be possible. From principal component analysis, it was found that selecting cotton introgression lines with higher lipid content may also significantly improve fatty acid composition which enhances the oil quality in cotton breeding. Besides that, hierarchical cluster analysis found that selecting cotton introgression lines from cluster 1 may be helpful to improve cottonseed oil content and quality. Among 590 cotton introgression lines, N1063 and N1190 from cluster 1 performed better in terms of higher oil and better fatty acid composition. However, validation of those superior lines is necessary to develop high-quality cotton varieties which is the suggestion for future studies. The results of this study would be helpful for tailoring the cotton plant for value-added seed properties to enhance the nutrient content of cottonseed. As this study is primarily focused on generating baseline information on the varietal performance and trait variation in a target environment, multi-location and multi-year trials which provide environmental stability and broader applicability are the limitation of our current study. So, the future research will expand the evaluation across multiple locations and multiple years to validate the consistency and robustness of the current findings.

## Figures and Tables

**Table 1:** Descriptive statistics for cottonseed oil content and fatty acids showing maximum, minimum, and average values in an introgression line population of Upland cotton.

Variable (%)	Mean	Minimum	Maximum
Lipid	24.01	10.71	43.10
Unsaturated fatty acids (USFA)	68.92	57.24	73.20
Saturated fatty acids (SFA)	30.92	27.20	42.76
Palmitic acids (C16:0)	24.36	20.30	30.00
Stearic acid (C18:0)	3.38	0.8	5.80
Oleic acid (C18:1)	17.12	13.39	20.40
Linoleic acid (C18:2)	51.04	39.44	56.10
Ratio of SFA to USFA	0.45	0.37	0.75

**Table 2:** Eigenvalues and principal components for oil and fatty acid content in cotton introgression lines.

Variables	PC I	PC II	PC III	PC IV	PC V	PC VI	PC VII
**Eigenvalue**	1.728	1.241	1.008	0.927	0.740	0.224	0.048
**Variability %**	0.427	0.220	0.145	0.123	0.078	0.006	0.001
**Cumulative %**	0.427	0.647	0.792	0.915	0.993	0.999	1.000

**Table 3:** Correlation between different variables and principal components.

Variables	PC I	PC II	PC III	PC IV	PC V	PC VI	PC VII
**Lipid %**	0.200	0.397	0.012	0.798	0.406	0.011	0.002
**C16:0 %**	−0.184	−0.563	0.370	−0.034	0.701	−0.136	−0.016
**C18:0 %**	0.045	0.578	−0.170	−0.576	0.548	0.050	0.002
**C18:1 %**	0.189	0.248	0.871	−0.110	−0.153	0.330	−0.007
**C18:2 %**	0.487	−0.342	−0.270	−0.028	0.138	0.744	−0.024
**SFA %**	−0.571	0.067	−0.031	0.092	0.022	0.415	0.698
**SFA/USFA**	0.570	0.090	−0.039	0.093	0.006	0.380	−0.716

**Table 4:** Mean value for each trait for individual clusters obtained from hierarchical cluster analysis.

Clusters	Lipid %	C16:0 %	C18:0 %	C18:1 %	C18:2 %	SFA %	SFA/USFA
1	32.51	23.44	3.73	17.53	51.33	29.97	0.43
2	24.31	23.84	2.98	16.55	53.06	29.59	0.42
3	19.58	26.51	2.49	16.03	51.87	31.66	0.46
4	20.60	24.12	3.63	16.41	45.29	36.38	0.57
5	21.79	23.87	4.18	16.67	50.51	31.22	0.46
6	25.68	23.20	3.59	16.72	48.81	32.90	0.49
7	20.91	24.56	4.04	17.51	52.67	29.45	0.42
8	23.34	23.92	2.95	18.42	51.94	29.11	0.41
9	16.92	26.86	2.96	16.72	48.73	33.97	0.51
10	23.28	25.30	2.98	17.94	49.97	31.41	0.46

## Data Availability

No new data were created or analyzed in this study.

## Supplementary Materials

The following supporting information can be downloaded at: https://www.mdpi.com/article/10.3390/plants14193078/s1, Table S1: List of cotton introgression lines in each cluster of dendrogam.

## Abbreviations

The following abbreviations are used in this manuscript:

USD	United States Dollar
MUFAs	Mono-unsaturated fatty acids
PUFAs	Poly-unsaturated fatty acids
SFAs	Saturated fatty acids
USFAs	Unsaturated fatty acids
ILs	Introgression Lines
FAME	Fatty Acid Methyl Ester
GC-FID	Gas Chromatography–Flame Ionization Detector
LDL	Low Density Lipoprotein
SOC	Seed Oil Content
PCA	Principal Component Analysis
