# Supplementary material for: Improvement of Cottonseed Oil and Fatty Acids Through Introgression Breeding in Upland Cotton
Source: Plants (Basel). 2025 Oct 5;14(19):3078. doi: 10.3390/plants14193078 (PMC12526194; doi:10.3390/plants14193078)
Supplement: Supplementary file 1 [file plants-14-03078-s001.zip › plants-3831778-supplementary.pdf]

Supplementary Information

Table S1: List of cotton introgression lines in each cluster of dendrogam

Cluster	Total count	Cotton Introgression lines
1	38	'N1056', 'N1057', 'N1062', 'N1063', 'N1073', 'N1085', 'N1089', 'N1093', 'N1099', 'N1122', 'N1137', 'N1141', 'N1183', 'N1184', 'N1188', 'N1190', 'N1194', 'N1196', 'N1202', 'N1206', 'N1216', 'N1217', 'N1228', 'N1229', 'N1234', 'N1235', 'N1238', 'N1239', 'N1245', 'N1246', 'N1248', 'N1319', 'N1368', 'N1428', 'N1463', 'N1557', 'N1564', 'N1584'
2	70	'N1066', 'N1070', 'N1071', 'N1077', 'N1078', 'N1081', 'N1083', 'N1087', 'N1097', 'N1136', 'N1140', 'N1144', 'N1146', 'N1147', 'N1175', 'N1177', 'N1178', 'N1181', 'N1182', 'N1186', 'N1193', 'N1200', 'N1201', 'N1213', 'N1226', 'N1230', 'N1232', 'N1236', 'N1237', 'N1240', 'N1251', 'N1252', 'N1267', 'N1307', 'N1309', 'N1310', 'N1311', 'N1313', 'N1314', 'N1316', 'N1317', 'N1321', 'N1322', 'N1323', 'N1324', 'N1327', 'N1363', 'N1423', 'N1427', 'N1466', 'N1551', 'N1558', 'N1561', 'N1566', 'N1567', 'N1568', 'N1570', 'N1573', 'N1575', 'N1577', 'N1578', 'N1579', 'N1580', 'N1582', 'N1583', 'N1585', 'N1586', 'N1588', 'N1589', 'N1590'
3	61	'N1001', 'N1035', 'N1036', 'N1037', 'N1039', 'N1040', 'N1042', 'N1043', 'N1044', 'N1045', 'N1046', 'N1047', 'N1048', 'N1049', 'N1050', 'N1052', 'N1053', 'N1055', 'N1058', 'N1059', 'N1061', 'N1065', 'N1067', 'N1069', 'N1072', 'N1074', 'N1075', 'N1076', 'N1079', 'N1080', 'N1082', 'N1084', 'N1086', 'N1088', 'N1090', 'N1091', 'N1092', 'N1094', 'N1095', 'N1096', 'N1098', 'N1100', 'N1118', 'N1139', 'N1205', 'N1210', 'N1214', 'N1222', 'N1247', 'N1277', 'N1283', 'N1284', 'N1287', 'N1288', 'N1289', 'N1291', 'N1292', 'N1297', 'N1304', 'N1315', 'N1329'
4	93	'L-129', 'L-130', 'L-131', 'L-132', 'L-136', 'N1106', 'N1197', 'N1209', 'N1212', 'N1215', 'N1225', 'N1231', 'N1233', 'N1241', 'N1242', 'N1249', 'N1250', 'N1253', 'N1254', 'N1255', 'N1256', 'N1257', 'N1259', 'N1260', 'N1261', 'N1262', 'N1263', 'N1264', 'N1265', 'N1268', 'N1269', 'N1271', 'N1273', 'N1274', 'N1276', 'N1278', 'N1279', 'N1280', 'N1282', 'N1285', 'N1286', 'N1290', 'N1293', 'N1294', 'N1295', 'N1298', 'N1299', 'N1300', 'N1302', 'N1308', 'N1318', 'N1325', 'N1331', 'N1332', 'N1334', 'N1336', 'N1337', 'N1338', 'N1339', 'N1340', 'N1341', 'N1342', 'N1343', 'N1344', 'N1345', 'N1346', 'N1377', 'N1391', 'N1415', 'N1416', 'N1419', 'N1420', 'N1429', 'N1431', 'N1437', 'N1438', 'N1444', 'N1464', 'N1479', 'N1482', 'N1485', 'N1486', 'N1488', 'N1542', 'N1545', 'N1548', 'N1549', 'N1552', 'N1553', 'N1565', 'N1569', 'N1576', 'N1604'
5	103	'L-107', 'L-109', 'L-111', 'L-112', 'L-125', 'L-133', 'L-140', 'L-98', 'N1041', 'N1051', 'N1220', 'N1223', 'N1306', 'N1330', 'N1333', 'N1335', 'N1347', 'N1351', 'N1352', 'N1354', 'N1356', 'N1358', 'N1359', 'N1361', 'N1362', 'N1364', 'N1370', 'N1371', 'N1372', 'N1373', 'N1376', 'N1382', 'N1384', 'N1388', 'N1392', 'N1393', 'N1394', 'N1396', 'N1398', 'N1399', 'N1400', 'N1402', 'N1404', 'N1407', 'N1408', 'N1410', 'N1412', 'N1413', 'N1414', 'N1417', 'N1418', 'N1421', 'N1422', 'N1424', 'N1426', 'N1430', 'N1433', 'N1434', 'N1435', 'N1436', 'N1439', 'N1440', 'N1441', 'N1442', 'N1445', 'N1450', 'N1451', 'N1452', 'N1453', 'N1454', 'N1455', 'N1456', 'N1457', 'N1458', 'N1460', 'N1461', 'N1465', 'N1467', 'N1468', 'N1469', 'N1474', 'N1476', 'N1477', 'N1489', 'N1493', 'N1494', 'N1543', 'N1544', 'N1546', 'N1547', 'N1554', 'N1555', 'N1556', 'N1559', 'N1562', 'N1571', 'N1572', 'N1574', 'N1607', 'N1608', 'N1611', 'N1612', 'N1616'

6	54	'L-102', 'L-103', 'L-105', 'L-106', 'L-108', 'L-110', 'L-113', 'L-116', 'L-117', 'L-118', 'L-119', 'L-120', 'L-121', 'L-122', 'L-123', 'L-124', 'L-126', 'L-127', 'L-128', 'L-135', 'L-137', 'L-139', 'L-96', 'L-97', 'L-99', 'N1064', 'N1385', 'N1386', 'N1387', 'N1395', 'N1406', 'N1447', 'N1459', 'N1472', 'N1473', 'N1475', 'N1480', 'N1481', 'N1484', 'N1487', 'N1491', 'N1492', 'N1538', 'N1539', 'N1550', 'N1591', 'N1595', 'N1599', 'N1600', 'N1601', 'N1603', 'N1609', 'N1613', 'N1615'
7	20	'L-100', 'L-104', 'L-114', 'L-134', 'L-138', 'N1038', 'N1068', 'N1159', 'N1348', 'N1349', 'N1350', 'N1389', 'N1401', 'N1446', 'N1448', 'N1449', 'N1462', 'N1483', 'N1605', 'N1610'
8	66	'N1002', 'N1003', 'N1004', 'N1005', 'N1006', 'N1015', 'N1023', 'N1029', 'N1030', 'N1054', 'N1102', 'N1103', 'N1116', 'N1117', 'N1120', 'N1124', 'N1126', 'N1127', 'N1128', 'N1130', 'N1131', 'N1132', 'N1142', 'N1148', 'N1150', 'N1153', 'N1155', 'N1156', 'N1158', 'N1160', 'N1172', 'N1176', 'N1179', 'N1211', 'N1218', 'N1227', 'N1244', 'N1266', 'N1270', 'N1275', 'N1281', 'N1296', 'N1320', 'N1353', 'N1355', 'N1357', 'N1360', 'N1365', 'N1366', 'N1374', 'N1378', 'N1379', 'N1383', 'N1390', 'N1397', 'N1403', 'N1409', 'N1411', 'N1443', 'N1470', 'N1478', 'N1495', 'N1536', 'N1541', 'N1598', 'N1617'
9	30	'N1101', 'N1104', 'N1105', 'N1110', 'N1111', 'N1113', 'N1143', 'N1145', 'N1162', 'N1169', 'N1170', 'N1173', 'N1174', 'N1185', 'N1187', 'N1189', 'N1191', 'N1192', 'N1195', 'N1198', 'N1199', 'N1203', 'N1204', 'N1305', 'N1326', 'N1375', 'N1405', 'N1425', 'N1560', 'N1563'
10	56	'N1007', 'N1008', 'N1009', 'N1010', 'N1012', 'N1013', 'N1014', 'N1016', 'N1017', 'N1018', 'N1019', 'N1020', 'N1021', 'N1022', 'N1024', 'N1025', 'N1026', 'N1031', 'N1032', 'N1033', 'N1034', 'N1060', 'N1107', 'N1108', 'N1109', 'N1112', 'N1114', 'N1115', 'N1119', 'N1121', 'N1125', 'N1129', 'N1133', 'N1134', 'N1135', 'N1138', 'N1149', 'N1151', 'N1152', 'N1154', 'N1157', 'N1161', 'N1171', 'N1180', 'N1207', 'N1208', 'N1219', 'N1221', 'N1224', 'N1243', 'N1258', 'N1301', 'N1303', 'N1328', 'N1432', 'N1540'
